# Using simulation-based training during hospital relocation: a controlled intervention study

**DOI:** 10.1186/s41077-022-00237-w

**Published:** 2022-12-16

**Authors:** Anders Lund Schram, Morten Søndergaard Lindhard, Magnus Bie, Maria Louise Gamborg, Neel Toxvig, Gitte Skov, Rune Dall Jensen

**Affiliations:** 1 Corporate HR, MidtSim, Central Denmark Region, Aarhus, Denmark; 2 Department of Paediatrics, Randers Regional Hospital, Randers, Denmark; 3 Department for Psychosis, Aarhus University Hospital-Psychiatry, Aarhus, Denmark

**Keywords:** In situ simulation, Hospital relocation, Sick leave, Readiness to perform, Uncertainty, Healthcare professionals

## Abstract

**Background:**

During hospital relocations, it is important to support healthcare professionals becoming familiar with new settings. Simulation-based training seems promising and in situ simulation has been suggested as a beneficial educational tool to prepare healthcare professionals for relocation. This study aimed to investigate the impact of a simulation-based training intervention on health professionals´ readiness to work in their new environment, as well as investigate sick leave before and after relocation.

**Methods:**

The study was a controlled intervention study implemented at a university hospital in Denmark. Simulation was used to prepare employees for workflows prior to relocation. Before relocation, 1199 healthcare professionals participated in the in situ simulation-based training program. Questionnaires on readiness to perform were distributed to participants at pre-, post-, and follow-up (6 months) measurement. In addition, data on participants’ sick leave was gathered from a business intelligence portal. To compare dependent and independent groups, paired and unpaired *t* tests were performed on mean score of readiness to perform and sick leave.

**Results:**

Compared to the control group, healthcare professionals participating in the intervention felt significantly more ready to work in a new hospital environment. As a measure of psychological wellbeing, register data indicated no difference in sick leave, when comparing intervention and control groups before and after participating in the in situ simulation-based training program.

**Conclusions:**

Healthcare professionals felt significantly more ready to work in a new environment, after participating in the in situ simulation-based training program, indicating that the intervention supported healthcare professionals during relocations. This may mitigate feelings of uncertainty; however, further research is needed to explore such effects.

**Trial registration:**

The study was approved by The Regional Ethics Committee (no. 1-16-02-222-22).

**Supplementary Information:**

The online version contains supplementary material available at 10.1186/s41077-022-00237-w.

## Introduction

Due to changing needs, modern healthcare services are constantly evolving [1–3]. To address such needs, many healthcare facilities are either being replaced or relocated [4, 5]. However, relocation of wards, sections, or entire hospitals may cause healthcare professionals to experience feelings of uncertainty due to substantial changes in workflow and care processes [6, 7]. Despite meticulous and detailed planning, relocation processes have been associated with negative consequences for both patients and healthcare professionals. Research shows that relocations can impact psychological wellbeing in terms of job satisfaction, job stress, and perceptions of service quality, leading to increased staff turnover and sick leave [8–13]. However, little is known about what tools can be used to mitigate such negative psychological impact. In this case, simulation-based training seems promising, as it can imitate a real-life scenario and prepare learners for new situations [7, 8, 14]. Hence, this study aimed to investigate the impact of a simulation-based training intervention on healthcare professionals´ readiness to perform in a new hospital, and rates of sick leave before and after relocation.

Using simulation in relocating processes is a fairly new approach. Brazil et al. (2019) argue that the effect of simulation interventions “ … lie in simulation shaping the culture and relationships that underpin and support structural or process specific interventions.” [15]. Simulation for system integration is an important additional feature when using simulation during relocation processes. Lin et al. (2016) demonstrated the importance of helping healthcare professionals familiarize with the relocation process and transition [7]. The study showed that inadequate preparation could negatively affect patient safety, and argued that dedicated time for orientation and simulation-based training could identify challenging areas and thereby increase safety for patients and healthcare staff [7]. This finding is supported by the other empirical studies, which emphasize the use of in situ simulation to prepare healthcare professionals for relocation [8, 9, 16–18]. Studies show that in situ simulation enhances healthcare professionals’ confidence and ability to care for patients in complex new settings [8, 16].

Brazil (2017) has argued for the term ‘translational simulation’, aiming to describe the use of simulation to improve performance through targeting specific healthcare processes or outcomes [19]. Thus, when relocating, system integration simulation has proven a feasible method of preparing staff for re-engineer and transferring quality of care [20]. Translation simulation has been used for ‘system probing’ and preparing staff, demonstrating how simulation can support cultural transmission both during onboarding processes and transitions [19, 21, 22] and identify latent safety threats [23]. A recent ethnographic study described how simulation can be used to onboard new staff members, transmitting the departmental culture through simulated scenarios [24]. Furthermore, when relocating hospital staff, translational simulation has also been argued as a feasible intervention for simulation as a system integration tool, including a particular impact on preparedness [25, 26]. However, this research focuses on organizational structures, more than psychological well-being of staff during relocation phases. In the present study, the main emphasis was on the wellbeing of the staff.

While research shows a need for training-interventions when planning for relocation of a hospital, no studies have, to our knowledge, investigated how such interventions impact psychological wellbeing, in terms of readiness to perform and related sick leave, amongst healthcare professionals [4, 16]. We sought to conduct a large, controlled simulation-based training intervention, to compare healthcare professionals’ readiness to perform in their new environment and investigate their sick leave before and after relocation of an entire hospital unit.

## Methods

The reporting guidance by Cheng et al. (2016), including key elements to report for simulation-based research, has been applied and is illustrated in Appendix 1 (Supplementary material) [27].

### Setting

The study was a controlled simulation-based intervention study implemented before the relocation of an independent psychiatric hospital unit, which was moved to a united University Hospital in Denmark in 2018.

### Participants

A total of 1711 healthcare professionals with patient contact working at the psychiatric wards and outpatient clinics, were recruited to participate in this study. Of these, 1199 participants took part in the in situ simulation-based training intervention, whereas the control group of 194 participants did not. The control group had no orientation and preparation prior to moving. Inclusion criteria for the intervention group were defined as healthcare professionals with patient contact that should be relocated. The control group consisted of employees unable to participate in the intervention.

### Intervention

Prior to the relocation of a larger psychiatric hospital in the Central Denmark Region, an in situ simulation-based training program was implemented and conducted between October 8th and November 2nd 2018. The aim of the program was to prepare clinical staff for working in a new hospital.

All simulation-based training sessions took place in the newly built hospital unit, and were completed two weeks before relocating, meaning that the facilities where the training took place were almost fully furnished but without patients. The newly built unit is a part of an 800-bed large university hospital. However, the location of the unit allowed the simulation-based intervention to take place without interfering with other hospital departments or patients.

In the planning phase of the intervention, the local hospital management identified three main research-based learning objectives:

1. Orientation: being able to find their way around in their own ward/clinic and gain insight into patient pathways in the new hospital.
2. Emergency calls: know the new routes for emergency assistance runs across the hospital, including the new alarm system and door locks.
3. Cardiac arrest: gain experience with basic life support (BLS) in case of a sudden heart attack in the new clinical setting.

The intervention was implemented in two steps. In the first step, a train-the-trainer program was implemented to educate 20 local healthcare professionals as facilitators of the simulations. The education of facilitators consisted of a 12-h course, consisting of lectures on the pedagogy and didactics of simulation and debriefing, and hands-on simulations. The facilitators were appointed by the local hospital administration based on teaching experience and professional background. The group of facilitators consisted of nurses, physiotherapists, doctors, healthcare assistants, psychologists, and occupational therapists, aiming to ensure an interprofessional group of facilitators consisting of representatives from all departments.

In the second step, the educated staff supported by experienced simulation educators facilitated the in situ simulation-based training program for the healthcare professionals. The training program lasted three hours. Firstly, participants were introduced to the new hospital and its layout including principles of navigation.

Secondly, an overall introduction to the learning objectives, methods, and duration of the three in-situ simulation sessions were conducted. Each session was conducted in groups of 10, and consisted of a briefing to the learning objectives and introduction to the differences between the old and new hospital facilities, followed by a scenario, ending with a facilitator-guided debriefing in three phases; reaction, analysis and application [28].

To ensure correct BLS treatment, an instructor certified in accordance with the European Resuscitation Council was present [29]. A Simulated manikin (Ambu International Ambu Man C). and a dispatcher-assisted defibrillator (Medtronic LIFEPAK CR Plus) was used in the cardiac arrest scenarios. The alarm system used at the new University Hospital was used in the emergency alarm scenarios. These were used on a predefined frequency in agreement with the hospital's alarm unit, hence the simulated emergency calls would not interfere with the code blue calls on the hospital. All the scenarios were designed as generic scenarios, focusing on the three learning objectives previously described. The overall course management was handled by two consultants, one specialized in simulation-based training and one with extensive local knowledge and direct contact to the local hospital management. The course curriculum is described in Appendix 2 (Supplementary material).

### Data collection and analysis

**Fig. 1:**
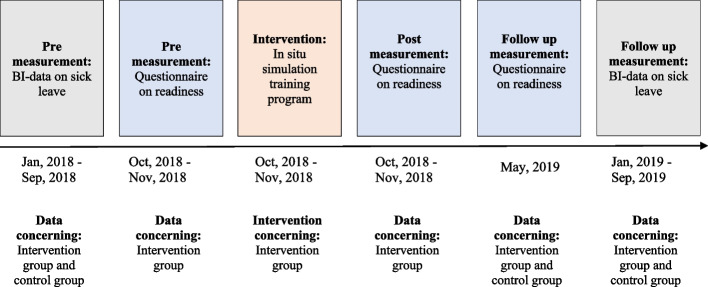
Overview of data collection. Illustration of timeline and types of data

In order to investigate effects of the in situ simulation-based training program, we conducted repeated questionnaire surveys, and utilized data from the Central Denmark Region Business Intelligence (BI) database. Figure 1 illustrates how and when data was collected. All analyses were performed using Stata/MP 17.0.

Through consultations with hospital administration, a questionnaire was developed by the authors of this study, based on current research on psychological and organizational outcomes [4, 6, 7, 16]. Questions were developed to meet practical needs when moving to a new setting, including readiness to perform in new facilities. Thus, the survey included three items related to readiness to perform, which were worded as follows: (1) ‘To what extent do you feel ready to navigate in your own ward/clinic?’, (2) ‘To what extent do you feel ready to participate in emergency calls at another ward/clinic?’ and (3) ‘To what extent do you feel ready handling emergency situations, for example a cardiac arrest?’. All items were measured using a Likert scale, consisting of (1) very ready, (2) ready, (3) neither nor, (4) not ready, or (5) not ready at all.

Furthermore, the questionnaire items collected background information, including; sex, age, profession, seniority, workplace, and an ID-number.

Questionnaire-data was collected as a pre-, post-, and follow-up measurement (Fig. 1). Pre-measurement was collected upon arrival to the new hospital facilities, prior to participating in the in situ simulation-based training program. Post-measurements were collected immediately after the in situ simulation-based training program. Pre- and post-measurements included employees participating in the simulation-based training program. The follow-up measurement was collected six months after the relocation, and included all employees working at the department of psychiatry.

All questionnaires were distributed using SurveyXact [30]. Pre- and post-measurements were collected by participants accessing the questionnaire through a QR-code with their smartphones as illustrated in Appendix 2 (Supplementary material). The follow-up measurement was collected by sending an email invitation to all employees at the Psychiatry Department at Aarhus University Hospital. Two additional reminder emails were sent, and posters reminding employees to answer the questionnaire were placed in common areas across the hospital.

Data from the questionnaires were categorized and analyzed separately in two groups, one consisting of ‘all participants’ (*n* = 1.199), and the other consisting of participants who completed all three surveys, which are characterized as the ‘complete case group’ (*n* = 143).

To investigate readiness, Likert scales were converted to range between 0 and 100, in which 1 was equal to 100, 2 = 75, 3 = 50, 4 = 25 and 5 = 0.

Business intelligence (BI) data were extracted from an ongoing administrative Human Resources database, covering all employment related information in the specific region. BI data was accessed by using the unique ID-number obtained from participants in the questionnaire. BI data included detailed individual sick leave registration, which was covered during two time periods, before and after the intervention, respectively. Sick leave was calculated by estimating the relationship between hours of absence and each staff’s proportion of a full-time position. Thus, part-time employment was taken into account. The first time period covered sick leave from January 30, 2018 until September 30, 2018. The second time period covered January 30, 2019 till September 30, 2019.

As in existing literature, analyses included mean scale scores and standard deviation [8, 16]. A change in mean scores indicated an improvement/aggravation in feeling ready and a higher/lower rate of sick leave. Paired sample *t* tests were applied to compare dependent groups over time, and non-paired sample *t* tests were used to compare across independent groups. Furthermore, non parametric tests including Wilcoxon signed-rank test (dependent data) and Wilcoxon rank sum test (independent data) were used to compare medians between groups. Finally, histograms in Appendix 3 (Supplementary material) was conducted to illustrate distribution of data.

## Results

### Participants

**Fig 2:**
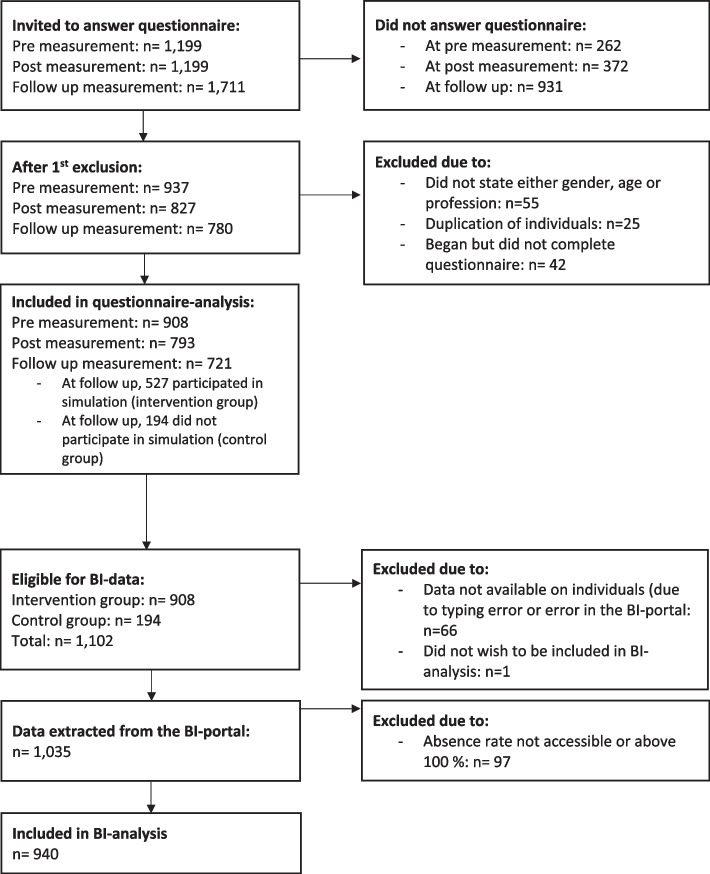
Flow diagram of the process of participant selection for analysis. Description of participation

In total, 1711 participants were invited to participate in the questionnaire. In the final analysis, 908, 793, and 721 participants were included at pre-, post-, and follow-up measurement, respectively. Overall, data on 1102 individuals were eligible to be gathered using the BI portal. Of these, 940 were included in the final analysis of sick leave. Details and reasons for exclusion can be found in Fig. 2 below.

Table 1 illustrates characteristics for intervention and control groups at pre-, post-, and follow-up measurements. Characteristics of the intervention group were similar across the three measurements. In comparison to the intervention group, the control group consisted of employees with lower mean age, less nurses, and less social and healthcare assistants, however, these differences were not significant.

**Table 1:** Sociodemographic characteristics at pre-, post-, and follow-up measurement

	Pre-measurement	Post-measurement	Follow-up measurement	Follow-up measurement	
				Participated in simulation	Did not participate in simulation (control group)
	*n* (%)				
**Participants**					
Participated/invited (%)	908/1199 (76)	793/1199 (69)	721/1711 (42)	527	194
**Age**					
20–30	155 (17)	144 (18)	127 (18)	70 (13)	57 (29)
30–40	264 (29)	235 (30)	204 (28)	144 (27)	60 (31)
40–50	192 (21)	169 (21)	164 (23)	122 (23)	42 (22)
50–60	203 (22)	172 (22)	158 (22)	134 (25)	24 (12)
> 60	94 (10)	73 (9)	68 (9)	57 (11)	11 (6)
**Gender**					
Female	711 (78)	618 (78)	591 (82)	423 (80)	168 (87)
Male	197 (22)	175 (22)	130 (18)	104 (20)	26 (13)
**Profession**					
Nurse	269 (30)	245 (31)	200 (28)	157 (30)	43 (22)
Doctor	128 (14)	121 (15)	83 (12)	63 (12)	20 (10)
Psychologist	148 (16)	121 (15)	116 (16)	90 (17)	26 (13)
Social and healthcare assistant	127 (14)	108 (14)	87 (12)	77 (15)	10 (5)
Pedagogue	73 (8)	65 (8)	44 (6)	35 (7)	9 (5)
Occupational therapist	38 (4)	30 (4)	23 (3)	19 (4)	4 (2)
Other^a^	125 (14)	103 (13)	168 (23)	86 (16)	82 (42)

Participants from questionnaire devided in age, gender, and profession

^a^Mainly; medical secretaries, service employees, or students

Change in readiness

Difference in readiness to perform was compared in two time periods (see Table 2). In total, 143 participants in the intervention group responded to all three surveys and were referred to as the ‘complete case group’.

**Table 2:** Readiness to perform before and after intervention

	Pre-measurement	Post-measurement	Follow-up measurement	Difference in mean score		
	Mean score (SD)	Mean score (SD)	Mean score (SD)	% difference^d^ (95% CI)	% difference^e^ (95% CI)	% difference for 'complete case group'^e^ (95% CI)
Theme	*n* = 908	*n* = 793	*n* = 527			*n* = 143
Feeling ready (navigation)^a^	41.5 (28.1)	70.4 (18.0)	79.6 (20.3)	28.8 (26.6; 31.2)	38.1 (35.4; 40.9)	34.4 (29.11; 39.8)*#
Feeling ready (emergency calls)^b^	28.9 (26.0)	61.5 (21.9)	58.2 (29.5)	32.5 (30.2; 34.8)	29.3 (26.3; 32.2)	30.4 (25.0; 35.8)*#
Feeling ready (cardiac arrest)^c^	42.8 (28.5)	72.1 (16.4)	64.2 (23.2)	29.3 (27.0; 31.5)	18.6 (15.9; 21.2)	22.0 (17.3; 26.8)*#

Mean score based on converted Likert scale

^a^Item: To what extent do you feel ready to navigate in your own ward/clinic?

^b^Item: To what extent do you feel ready to participate in emergency calls at another ward/clinic?

^c^Item: To what extent do you feel ready handling emergency situations (e.g., a cardiac arrest)?

^d^Difference in mean score from pre-measurement to post-measurement

^e^Difference in mean score from pre-measurement to follow-up measurement

*Indicates a statistically significant difference over time (*p* < 0.005) (based on paired sample)

^#^Indicates a statistically significant difference (Wilcoxon signed-rank test) in medians between dependent groups (*p* < 0.005)

Among all participants, a statistically significant increase of 28.8 to 32.5% was observed in all three mean scores from before the in situ simulation-based training program (pre-measurement) to after the training program (post-measurement).

Difference in mean score from pre-measurement to follow-up measurement was compared. Here, the mean score increased within the theme ‘feeling ready to navigate in your own ward/clinic’. However, the mean scores regressed in the remaining two other themes, ‘feeling ready to participate in emergency calls’ and ‘feeling ready handling emergency situations (e.g., a cardiac arrest)’.

Additionally, we compared differences in mean scores from pre measurement to follow-up measurement among the ‘complete case group’, representing participants that responded to all questionnaires. Mean scores in all themes improved statistically significantly in the complete case group, and were comparable to mean scores among all participants. The non-parametric Wilcoxon signed-rank test also supported a significant difference (*p* < 0.05) in all dependent mean scores.

We compared readiness to perform among employees participating in the training program with the control group (Table 3). Compared to employees not participating in the simulation-based training, we observed a statistically significant difference (*p* < 0.05) over time in all mean scores, including 6.1% (2.6; 9.5) higher mean score in ‘feeling ready to navigate in your own section’, a 18.7% (13.7;23.7) higher mean score in ‘feeling ready to participate in emergency calls’, and a 10.3% (6.3;14.4) higher mean score in ‘feeling ready handling emergency situations (e.g., a cardiac arrest)’ in the intervention group. Non parametric Wilcoxon rank sum tests also supported a significant difference (*p* < 0.05) in all independent mean scores.

**Table 3:** Readiness to perform in intervention and control group at follow-up survey

	Follow-up survey		
	Intervention group (simulation)	Control group (no simulation)	
	Mean score (SD)	Mean score (SD)	Difference^d^ (95% CI)
Theme	*n* = 527	*n* = 194	
Feeling ready (navigation)^a^	79.6 (20.3)	73.6 (22.9)	6.1 (2.6; 9.5)*#
Feeling ready (emergency calls)^b^	58.2 (29.5)	39.4 (32.2)	18.7 (13.7; 23.7)*#
Feeling ready (cardiac arrest)^c^	64.2 (23.2)	53.9 (27.6)	10.3 (6.3; 14.4)*#

Mean score based on converted Likert scale

^a^Item: To what extent do you feel ready to navigate in your own ward/clinic?

^b^Item: To what extent do you feel ready to participate in emergency calls at another ward/clinic?

^c^Item: To what extent do you feel ready handling emergency situations (e.g., a cardiac arrest)?

^d^Difference depending on participation in simulation

*Indicates a statistically significant difference (*p* < 0.005) (Based on unpaired sample *t* test)

^#^Indicates a statistically significant difference (Wilcoxon rank sum test) in medians between independent groups (*p* < 0.005)

### Change in sick leave

Table 4 compares sick leave (rate of absence) between groups before and after the training program and the relocation of the Department of Psychiatry. We observed a significant increase in the rate of absence from the first time period to the second time period by 2.1% (1.2; 2.9) among all participants. Similarly, when looking at the intervention group, the rate of absence increased by 2.1% (1.2; 3.1), and in the control group it increased by 1.6% (− 0.6; 3.8), though not statistically significant.

**Table 4:** Sick leave before and after in situ simulation training program

	All participants	Intervention group	Control group	
	Mean (SD)	Mean (SD)	Mean (SD)	Difference in mean score when comparing groups: % (CI)^c^
	*n* = 940	*n* = 802	*n* = 138	
Rate of absence % (first time period)^a^	3.7 (7.9)	3.8 (7.8)	3.1 (7.1)	0.7 (− 0.7 ; 2.1)
Rate of absence % (second time period)^b^	5.8 (11.9)	6.0 (12.2)	4.7 (10.1)	1.3 (− 0.9; 3.4)#
	Mean (CI)	Mean (CI)	Mean (CI)	Difference in mean score when comparing groups over time: % (CI)^c^
Change in absence over time: % (CI)^d^	2.1 (1.2; 2.9)*	2.2 (1.2; 3.1)*	1.6 (− 0.6 ; 3.8)	0.5 (− 3.0; 1.9)^e^

Rate of absence in percentage devided in intervention and control group

*Note* ^a^ Time period: January, 2018 till October, 2018

^b^January, 2019 till October, 2019

^c^Unpaired sample *t* test

^d^Paired sample *t* test

^e^Difference in mean score in the intervention group and the control group over time

*Indicates a statistically significant difference (*p* < 0.05) (based on unpaired sample *t* test)

^#^Indicates a statistically significant difference (Wilcoxon rank sum test) in medians between independent groups (*p* < 0.005)

Furthermore, change in rate of absence across groups was compared. Before the intervention, the rate of absence in the intervention group was 0.7% higher, when compared to the control group, though not significantly. After intervention, the rate of absence in the intervention group was 1.3% higher, when compared to the control group, likewise not significantly. Finally, we compared the rate of absence in the intervention group and the control group over time. Based on unpaired sample *t* tests, no significant difference was found. As in the unpaired sample *t* test, non parametric Wilcoxon rank sum tests showed no significant difference (*p* < 0.05) in mean scores in the second time period. However, unlike the unpaired sample *t* test, Wilcoxon rank sum tests showed significant difference (*p* < 0.05) in mean scores in the first time period.

## Discussion

This intervention study investigated the influence of simulation-based training, aiming to prepare healthcare staff for a hospital relocation. After participating in an in situ simulation-based training program, findings showed that employees felt significantly more ready to work in a new hospital environment. Table 2 illustrated an improvement in readiness after both the training program (post-measurement) and after the move into new facilities (follow-up measurement).

However, it is unknown if this difference in readiness at the follow-up measurement is due to the in situ simulation program, or the fact that employees have been working in the new facilities for 6 months. Table 3 showed a statistically significant difference in readiness to perform in new facilities, when compared to the control group. Thus, employees participating in the training program considered themselves to be more ready to perform in new facilities, when compared to employees not participating in the training program, indicating an experienced effect of participating in preparatory simulation training.

BI data though, indicated no difference in sick leave, when comparing intervention and control groups. In fact, the rate of absence increased significantly among all employees after the relocation.

Knowing the intricacies of what facilitate positive outcomes from simulation interventions can be difficult, as intervention and context can differ [15]. Similar issues might be relevant in this study, as we did not find any effect on sick leave between groups. While we saw an effect on readiness both at post-test and follow-up, it is still unclear which specific factors led to this result. As previously mentioned, the relocation of a hospital is associated with an increase in adverse health-related outcomes including stress and sick leave among healthcare professionals [9, 11–13, 31]. Despite previous studies arguing that pre-relocation simulation-based training could accommodate potential stressors, this was not supported by findings in this study [11–13]. Accordingly, contrary to a previous study which found that sick leave was reduced after a simulation-based training intervention, we found an increase in sick leave after the simulation-based training and the hospital relocation in both the intervention and control group [12]. The differences between the findings may reflect that the present study investigated sick leave before and after a hospital relocation, whereas the study by Meurling and colleagues examined the relationship between simulation-based team training and self-efficacy [12]. This discrepancy may indicate that while simulation-based interventions in themselves can impact psychological wellbeing and reduce sick leave, major changes such as relocations have a more complex influence on employee’s psychological wellbeing. That being said, it would be reasonable to speculate that increased readiness would impact subjective psychological wellbeing, by accommodating related uncertainty, and decreasing stress levels. As sick leave and burnout among healthcare professions are often associated with stress, this field of research is defined as complex and difficult to measure [13, 32]. Future research including qualitative methods would be relevant.

While some research demonstrates that simulation can be used to prepare employees for hospital relocation, indicating that simulation-based training can be used to prepare employees before moving to a new hospital, such studies are still sparse [8, 12, 16, 18]. This study adds a large-scale intervention, supporting these results, as we found that employees felt significantly more ready to work in a new hospital environment, after participating in an in situ simulation-based training program. Thus, there is increasing evidence for the importance of using in situ simulation as an integral part of relocation processes. It would be of relevance to investigate more patient related outcomes in relation to both relocation and the influence of in situ simulation programs.

### Strengths and limitations

This study included 1102 respondents, including 908 participants in the intervention group and 194 participants in the control group. The number of participants is considerably higher than previously published simulation-based studies, including between 28 and 151 respondents [8, 12, 16, 17]. The higher number of participants in this study reduces the risk of type I and II errors, lowering the risk of either accepting a false hypothesis or rejecting a true hypothesis [33]. Furthermore, the present study included a control group at the follow-up measurement, which to our knowledge is unique, when compared to existing literature [8, 16–18]. The prospective and repeated collection of data allowed for comparisons on readiness to perform and sick leave over time.

For employees at pre- and post-measurement, response rate varied between 69 and 76% across measurements. The high response rate at pre- and post-measurements is considered a strength. The response rate at follow-up measurement however, was at only 42%, which may be considered low, leading to a limitation in the present study. A reason for the low response rate is that every employee not participating in the intervention in the Department of Psychiatry, was invited to participate in the follow-up measurement. Lastly, the questionnaire was not a validated psychometric tool, leading to a risk of potential errors in measurements [34]. However, bias would most likely be non-differential, which is not severe, since the influence would be identical across compared groups [35].

### Data analysis

Data in this study was analyzed by using a mean score. Applying mean scores, polarized and nuanced distribution of data might be missed. By performing Wilcoxon signed-rank (dependent data) and Wilcoxon rank sum (independent data) tests, no assumption of data having a normal distribution occurs. In Table 2 and Table 3, *p* values less than 0.05 supported existing findings, showing a significant difference between groups. Although, only one of two *p* values in Table 4 supported difference in rate of absence, leading to inconsistency between groups. Using unpaired *t* test, we found no significant difference between groups in the first time period. However, a Wilcoxon rank sum test showed significant difference (*p* < 0.005) between groups in this time period. Histograms in Appendix 3 (Supplementary material) covering data from Tables 2 and 3 showed a normal distribution, whereas histograms covering data from Table 4 showed a right-sided distribution of data. This not normal distribution of data from Table 4 was expected, since the rate of sick leave could not be less than zero percentage, meaning the proportion of healthcare professionals with a high rate of sick leave would influence the distribution of data. Thus, the distribuition of data was somewhat as expected.

### Intervention

The intervention consisted of a three-hour simulation-based training session 2 weeks before the relocation. For the majority of the participants, simulation was a new learning modality prior to the intervention and thereby, might have been an unfamiliar method and concept among many employees. The fact that employees had little experience with simulation, might have influenced or limited the benefits of the training program. Furthermore, since simulation was implemented in a not yet fully utilized department, it might have made it difficult to recreate an everyday reality and thus, mirror authentic situations, making it harder for the participants to immerse themselves in the scenarios [14]. Based on this study it would be interesting to investigate if the simulation intervention would be better suited soon after the relocation. This would however be complicated by the presence of patients.

Another interesting viewpoint, having this large sample of healthcare staff, would be a large-scale translation simulation intervention study, investigating system failures in relation to specific healthcare outcomes [19].

## Conclusion

This study investigated readiness to perform and sick leave, before and after a hospital relocation and a simulation-based training intervention. Employees felt significantly more ready to work in a new hospital environment, after participating in the in situ simulation-based training program. Thus, this study supports the use of simulation in relocation processes. However, sick leave increased among all employees six months after the move, indicating that relocation phases have psychological impact on employees, which need more qualitative investigation in future research.

## Supplementary Information

**Additional file 1: Appendix 1.** Key Elements to Report for Simulation-Based Research – adapted from Cheng et al. (2016) [27].

**Additional file 2: Appendix 2.** Course curriculum.

**Additional file 3: Appendix 3.** Histograms of data distribution.

## Data Availability

The datasets used and/or analysed during the current study are available from the corresponding author on reasonable request.

## Abbreviations

BI: Business intelligence
BLS: Basic life support
SD: Standard deviation
CI: Confidence interval
