# Supplementary material for: Using simulation-based training during hospital relocation: a controlled intervention study
Source: Adv Simul (Lond). 2022 Dec 16;7:41. doi: 10.1186/s41077-022-00237-w (PMC9758894; doi:10.1186/s41077-022-00237-w)
Supplement: Supplementary file 1 — Additional file 1: Appendix 1. Key Elements to Report for Simulation-Based Research – adapted from Cheng et al. (2016) [27]. [file 41077_2022_237_MOESM1_ESM.docx]

# Supplementary material

**Appendix 1**: Key Elements to Report for Simulation-Based Research – adapted from Cheng et al. (2016) [27].

| **Elements** | **Sub-elements** | **Descriptor** | **Response** |
| --- | --- | --- | --- |
| Participant orientation | Orientation to the simulator | Describe how participants were oriented to the simulator (e.g., method, content, duration). | See Page 6/7:  The training program lasted three hours. First, participants were introduced to the new hospital and its structural layout, including a walk-through of the building to ease the individual indoor navigation. |
|  | Orientation to the environment | Describe how participants were oriented to the environment (e.g., method, content, duration). | See page 7:  Secondly, an overall introduction to the learning objectives, methods, and duration of the three in-situ simulation sessions were conducted. Each session was conducted in groups of 10, and consisted of a briefing to the learning objectives and introduction to the differences between the old and new hospital facilities, followed by a scenario, ending with a facilitator-guided debriefing in three phases; reaction, analysis and application |
| Simulator type | Simulator make and model | Describe the simulator make and model. | See page 7:  A Simulated manikin (Ambu International Ambu Man C). and a dispatcher-assisted defibrillator (Medtronic LIFEPAK CR Plus) was used in the cardiac arrest scenarios |
|  | Simulator functionality | Describe functionality and/or technical specifications that are relevant to the research question. Describe modifications, if any. Describe limitations of the simulator. | Data from the simulations were not used for debriefing or in the data collection. |
| Simulation environment | Location & Equipment | Describe where the simulation was conducted (e.g., in situ clinical environment, simulation center, etc.). | See page 6:  All simulation-based training sessions took place in the newly built hospital unit, and were completed two weeks before relocating, meaning that the facilities where the training took place were almost fully furnished but without patients. |
|  | Equipment | Describe the nature of the equipment available (e.g., type, amount, location, size, etc.). | A simulated manikin and a dispatcher-assisted defibrillator was used in the cardiac arrest scenario. |
|  | External stimuli | Describe any external stimuli (e.g., background noise). | See page 6;  The newly built unit is a part of an 800-bed large university hospital. However, the location of the unit allowed the simulation-based intervention to take place without interfering with other hospital departments or patients. |
| Simulation event/scenario | Event description | Describe if the event was programmed and/or scripted (e.g., orientation to event, scenario progression, triggers). If a scenario was used, the scenario script should be provided as an appendix. | See appendix 2 |
|  | Learning objectives | List the learning objectives and describe how they were incorporated into the event. | See page 6:  In the planning phase of the intervention, the local hospital management identified three main research-based learning objectives:  1) Orientation: Being able to find their way around in their own ward/clinic and gain insight into patient pathways within the new hospital.  2) Emergency calls: Get to know the new routes for emergency assistance runs across the hospital, including the new alarm system and door locks.  3) Cardiac arrest: Gain experience with basic life support (BLS) in case of cardiac arrest in the new clinical setting. |
|  | Group vs. individual practice | Describe if the simulation was conducted in groups or as individuals. | See page 7:  … the simulation sessions were conducted in groups of 10 |
|  | Use of adjuncts | Describe if adjuncts (e.g., moulage, media, props) were used. | No adjuncts were used |
|  | Facilitator/operator characteristics | Describe experience (e.g., clinical, educational), training (e.g., fellowship, courses), profession. | See page 6-7  … a train-the-trainer program was implemented to educate 20 local healthcare professionals as facilitators of the simulations. The education of facilitators consisted of a 12 hour course, consisting of lectures on the pedagogy and didactics of simulation and debriefing, and hands-on simulations. The facilitators were appointed by the local hospital administration based on teaching experience and professional background. The group of facilitators consisted of nurses, physiotherapists, doctors, healthcare assistants, psychologists and occupational therapists, aiming to ensure an interprofessional group of facilitators consisting of representatives from all departments.  An instructor, certified in accordance with the European Resuscitation Council was present during the cardiac arrest scenario.  The experienced simulation instructors supporting the facilitators were all educated with simulation and worked full-time at a simulation center. |
|  | Pilot testing | Describe if pilot testing was conducted (e.g., number, duration, frequency). | After the train the trainer program, the three scenarios were tested using facilitators as participants. |
|  | Actors/confederates/standardized/simulated patients | Describe experience (e.g., clinical, educational), training (e.g., fellowship, courses), profession, sex. Describe various roles, including training, scripting, orientation, and compliance with roles. | No Actors/confederates/standardized/simulated patients were used |
| Instructional design (for educational interventions) or exposure (for simulation as investigative methodology) | Duration | Describe the duration of the educational intervention. If the intervention involves more than one segment, describe the duration of each segment. | The intervention lasted three hours for each group of participants. This was divided into 30 mins to introduction, 45 mins of each scenario including briefing, simulation and debriefing, and 15 mins follow-up and conclusion. |
|  | Timing | Describe the timing of the educational intervention relative to the time when assessment/data collection occurs (e.g., just-in-time training). | See page 8:  Questionnaire-data was collected as a pre-, post-, and follow-up measurement (Figure 1). Pre measurement was collected upon arrival to the new hospital facilities, prior to participating in the in situ simulation-based training program. Post measurements were collected immediately after the in situ simulation-based training program. Pre- and post-measurements included employees participating in the simulation-based training program. The follow up measurement was collected six months after the relocation, and included all employees working at the department of psychiatry.  See page 9:  Business intelligence (BI) data were extracted from an ongoing administrative Human Resources database, covering all employment related information in the specific region |
|  | Frequency/repetitions | Describe how many repetitions were permitted and/or the frequency of training (e.g., deliberate practice). | Each scenario allowed to 2-3 repetitions of the case. |
|  | Clinical variation | Describe the variation in clinical context (e.g., multiple different patient scenarios). | All scenarios were conducted in the same clinical context in the newly built hospital unit. |
|  | Standards/assessment | Describe predefined standards for participant performance (e.g., mastery learning) and how these standards were established. | No assessment of the participants’ performance was conducted, however all scenarios ended with a structured debriefing including rooms for improvements. |
|  | Adaptability of intervention | Describe how the training was responsive to individual learner needs (e.g., individualized learning). | See page 6:  In the planning phase of the intervention, the local hospital management identified three main research-based learning objectives:  1) Orientation: Being able to find their way around in their own ward/clinic and gain insight into patient pathways in the new hospital.  2) Emergency calls: Know the new routes for emergency assistance runs across the hospital, including the new alarm system and door locks.  3) Cardiac arrest: Gain experience with basic life support (BLS) in case of a sudden cardiac arrest in the new clinical setting. |
|  | Range of difficulty | Describe the variation in difficulty or complexity of the task. | Learning goals were identified by the hospital management and adjusted by the experienced simulation instructors and facilitators in order to fit the needs of the participants. |
|  | Non-simulation interventions and adjuncts | Describe all other non-simulation interventions (e.g., lecture, small group discussion) or educational adjuncts (e.g., educational video), how they were used, and when they were used relative to the simulation intervention. | No non-simulation interventions were used. |
|  | Integration | Describe how the intervention was integrated into curriculum. | No curriculum for the re-location were made beside this intervention |
| Feedback and/or debriefing | Source | Describe the source of feedback (e.g., computer, simulator, facilitator). | The facilitators and the experienced simulation instructors provided feedback to the participants. |
|  | Duration | Describe the amount of time spent. | Each scenario including 10-15 minutes of debriefing. |
|  | Facilitator presence | Describe if a facilitator was present (yes/no), and if so, how many facilitators. | Yes. 1 facilitator and 1 experienced simulation instructor were present during all scenarios. In addition, an instructor certified in accordance with the European Resuscitation Council was present during the cardiac arrest scenario |
|  | Facilitator characteristics | Describe experience (e.g., clinical, educational), training (e.g., fellowship, courses), profession, sex. | See page 6:  The education of facilitators consisted of a 12 hour course, consisting of lectures on the pedagogy and didactics of simulation and debriefing, and hands-on simulations. The facilitators were appointed by the local hospital administration based on teaching experience and professional background. The group of facilitators consisted of nurses, physiotherapists, doctors, healthcare assistants, psychologists and occupational therapists, aiming to ensure an interprofessional group of facilitators consisting of representatives from all departments.  An instructor certified in accordance with the European Resuscitation Council was present  The experienced simulation instructors supporting the facilitators were all educated within simulation and employed full-time at a simulation center. |
|  | Content | Describe content (e.g., teamwork, clinical, technical skills, and/or inclusion of quantitative data, etc.). | The content in scenarios consisted of training teamwork skills during emergency calls and cardiac arrest, and technical skills within way-finding and basic life support. |
|  | Structure/method | Describe the method of debriefing/feedback and debriefing framework used (ie, phases). | See page 7:  Ending with a facilitator-guided debriefing in three phases; reaction, analysis and application [28]. |
|  | Timing | Describe when the feedback and/or debriefing was conducted relative to the simulation event (e.g., terminal vs. concurrent). | See page 7:  Each session was conducted in groups of 10, and consisted of a briefing to the learning objectives and introduction to the differences between the old and new hospital facilities, followed by a scenario, ending with a facilitator-guided debriefing… [28] |
|  | Video | Describe if video was used (yes/no) and how it was used. | No video was used |
|  | Scripting | Describe if a script was used (yes/no) and provide script details as an appendix. | See appendix 1 |
