# Supplementary material for: Using simulation-based training during hospital relocation: a controlled intervention study
Source: Adv Simul (Lond). 2022 Dec 16;7:41. doi: 10.1186/s41077-022-00237-w (PMC9758894; doi:10.1186/s41077-022-00237-w)
Supplement: Supplementary file 2 — Additional file 2: Appendix 2. Course curriculum. [file 41077_2022_237_MOESM2_ESM.docx]

# Supplementary material

| **Appendix 2:** Course curriculum | | | | |
| --- | --- | --- | --- | --- |
|  | **Duration in minutes** | **Activity** | **Facilitator/ participant ratio** | **Structure** |
| Before training | 10 | Bus departure from old hospital | 0/60 |  |
|  | 15 | Joint welcome and introduction to the new setting | 1/60 |  |
|  | 15 | Brief tour in the facility focusing on the layout | 1/30 |  |
| In situ simulation training program | 45 | 1st round in situ simulation including navigation at own ward/clinic | 1/10 | Structure: Briefing (10-15 min) / Simulation (20-25 min) / Debriefing (10-15 min) |
|  | 45 | 2nd round in situ simulation including navigation between wards/clinics and an introduction to the new alarm system | 1/10 |  |
|  | 45 | 3rd round in situ simulation including BLS training | 2/10 |  |
| After training | 15 | Joint summary and questions | 1/60 |  |
|  | 10 | Bus departure to the old hospital | 0/60 |  |
