# Supplementary material for: Using simulation-based training during hospital relocation: a controlled intervention study
Source: Adv Simul (Lond). 2022 Dec 16;7:41. doi: 10.1186/s41077-022-00237-w (PMC9758894; doi:10.1186/s41077-022-00237-w)

# Supplementary material

**Appendix 3:** Histograms of data distribution

Data distribution of ‘Readiness to perform’ at pre measurements:


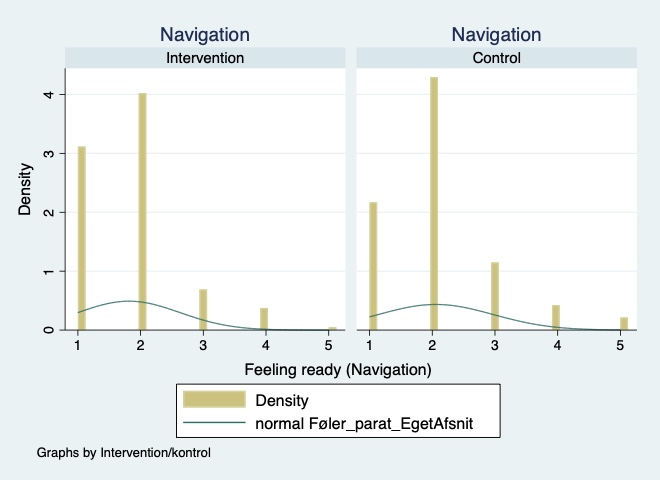

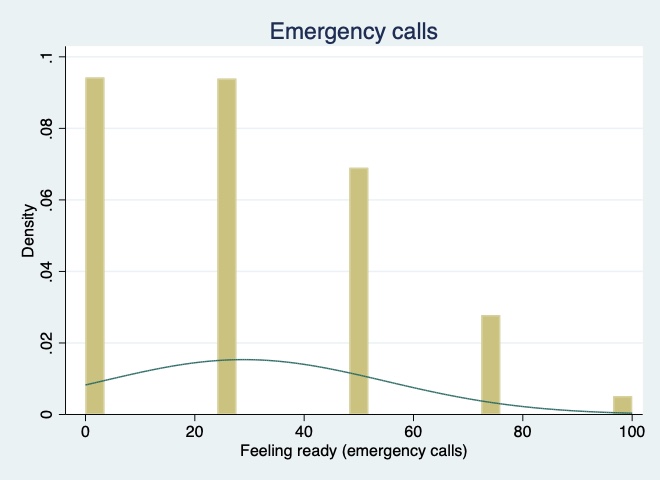

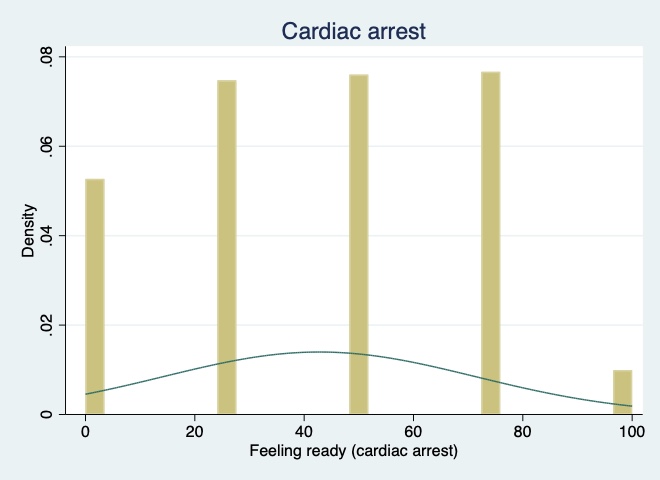


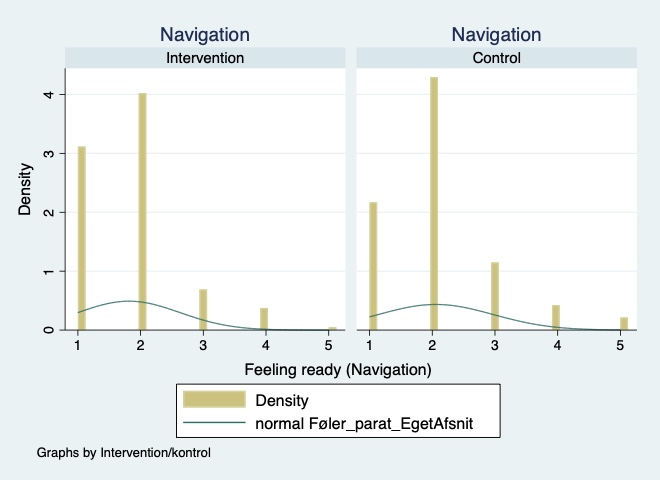

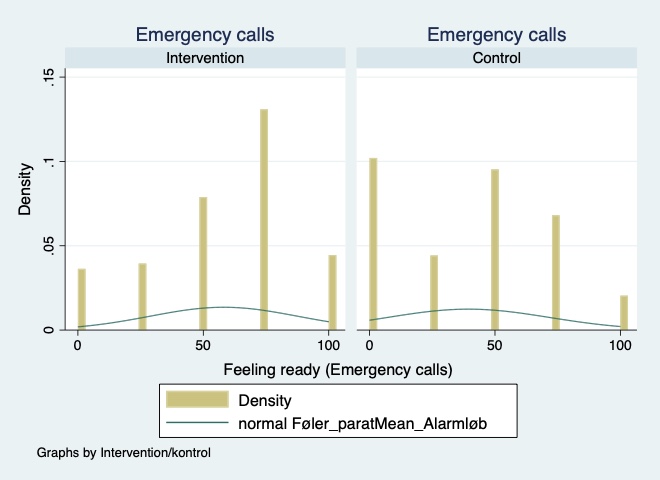

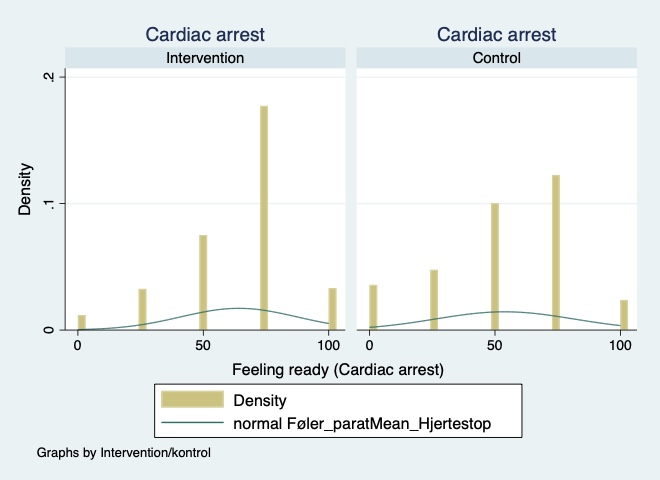


Data distribution of ‘Sick leave’ at two time periods:


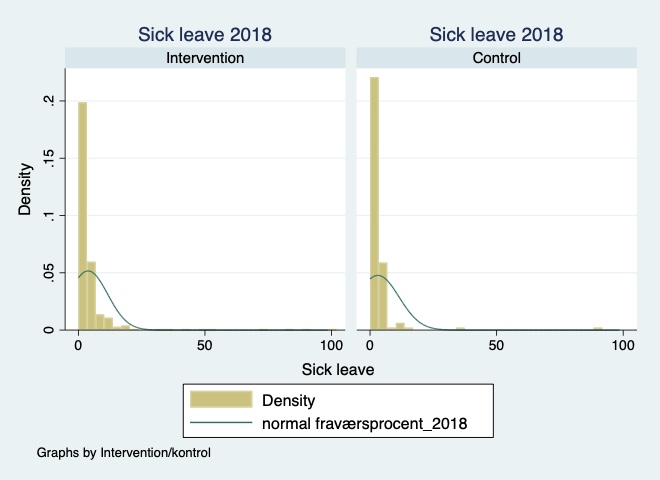

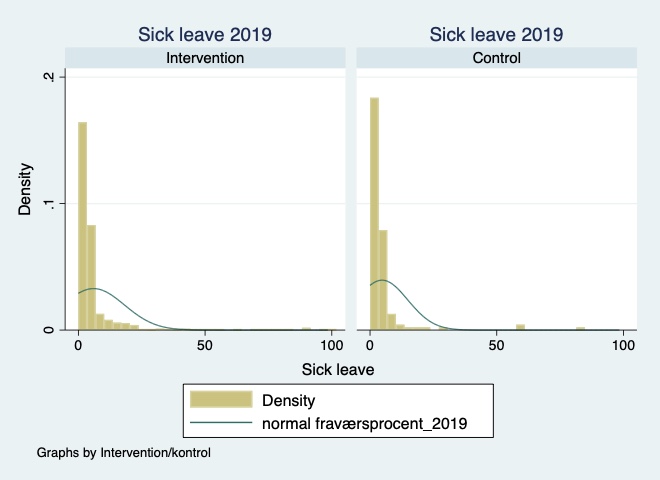

Supplement: Supplementary file 3 — Additional file 3: Appendix 3. Histograms of data distribution. [file 41077_2022_237_MOESM3_ESM.docx]
